# Splenic and Peritoneal Metastases with Para-aortic and Virchow Lymph Node Metastases: Late Recurrence of Ovarian Cancer 30 Years after Initial Treatment

**DOI:** 10.31662/jmaj.2021-0062

**Published:** 2021-09-01

**Authors:** Kosei Takagi, Takahito Yagi, Toshiyoshi Fujiwara

**Affiliations:** 1 Department of Gastroenterological Surgery, Okayama University Graduate School of Medicine, Dentistry, and Pharmaceutical Sciences, Okayama, Japan

**Keywords:** Ovarian cancer, late recurrence, splenic metastasis

A 76-year-old woman presented with abdominal pain. The positron emission tomography (PET)/computed tomography (CT) revealed a large splenic tumor and peritoneal dissemination with para-aortic and Virchow lymphadenopathy, which were initially suspected of malignant lymphoma (Figure 1). Prior to further examination, the patient developed a mechanical bowel obstruction, requiring surgery with partial resection of the intestine (Figure 2). The patient had history of ovarian cancer treated with radical total hysterectomy and bilateral adnexectomy followed by adjuvant chemotherapy 30 years ago. Although no recurrence was observed during long-term follow-up, pathological findings of the resected intestine showed high-grade serous carcinoma (Figure 3). Immunohistochemically, the tumor cells were positive for PAX8, WT-1, and p53 and negative for TTF-1, CDX-2, mammaglobin, and GCDFP-15. Therefore, the patient was diagnosed with late recurrence of ovarian serous carcinoma. Thus far, only six cases with late recurrence, after 20 years, of ovarian cancer treated via surgery and chemotherapy have been reported ^(1), (2)^; however, this case is the first to demonstrate a recurrence of ovarian cancer more than 30 years after the initial treatment. Cancer stem cell might be associated with this recurrence ^(2), (3)^.

**Figure 1.**
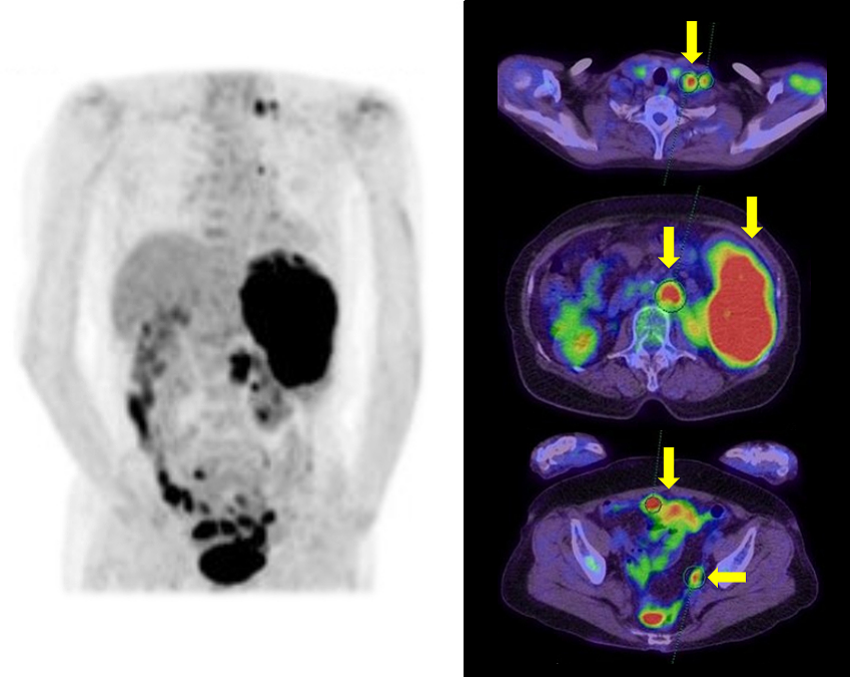
Positron emission tomography (PET)/computed tomography (CT) showed abnormal accumulation in the spleen, the para-aortic and Virchow’s lymph node, the intestine, and the pouch of Douglas (arrows).

**Figure 2.**
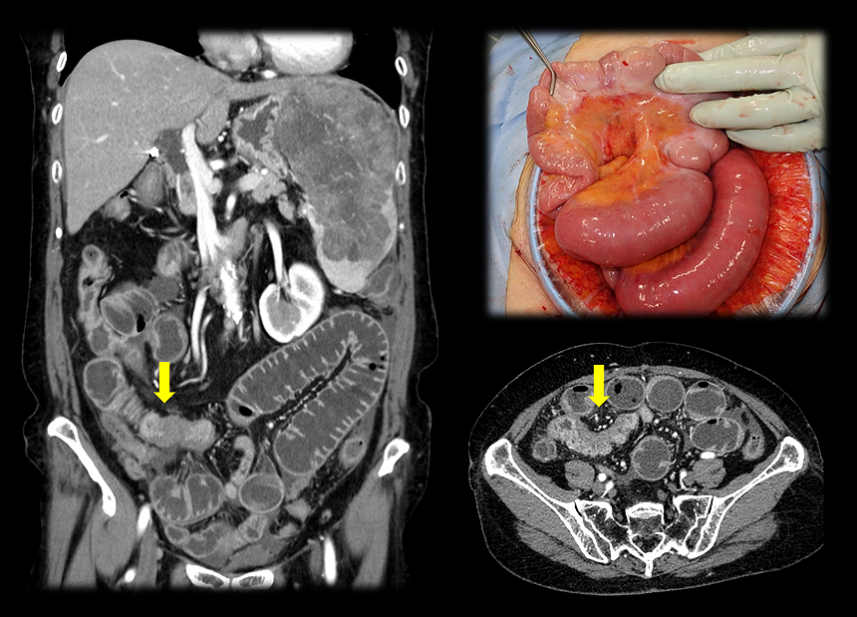
Computed tomography revealed small bowel wall thickening (arrow), which caused a mechanical bowel obstruction. At surgery, peritoneal dissemination and ascites were confirmed. The patient underwent a partial resection of the intestine.

**Figure 3.**
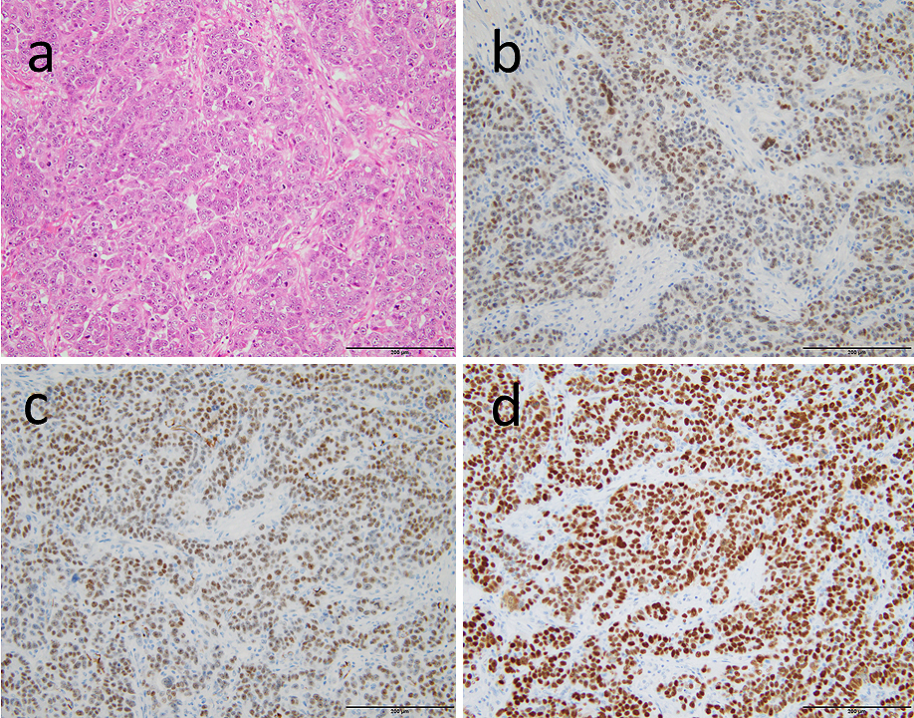
Pathological examination of the resected intestine showing features of high-grade serous carcinoma characterized by papillary and solid growth patterns (a). Immunohistochemical analysis showing positive staining for the PAX8 (b), WT-1 (c), and p53 (d).

## Article Information

### Conflicts of Interest

None

### Author Contributions

KT wrote the draft. All authors contributed equally to the manuscript. All authors edited, read, and approved the final manuscript.

### Approval by Institutional Review Board (IRB)

Not applicable.

### Informed Consent

Written informed consent was obtained from the patient for publication of this report and any accompanying images.
